# *In-vitro* bioaccessibility and bioavailability of heavy metals in mineral clay complex used in natural health products

**DOI:** 10.1038/s41598-020-65449-4

**Published:** 2020-06-01

**Authors:** Xiumin Chen, Anika Singh, David D. Kitts

**Affiliations:** 1 Food, Nutrition, and Health, Faculty of Land & Food Systems. 2205 East Mall, University of British Columbia, Vancouver, BC V6T 1Z4 Canada; 2 School of Food and Biological Engineering, Jiangsu University, Zhenjiang, Jiangsu 212013 P.R. China

**Keywords:** Environmental sciences, Chemistry

## Abstract

Commercial mineral clays that claim to have healing properties are also known to contain trace amounts of heavy metals, albeit the risk of consuming many of them is not entirely known. The primary objective of this study was to evaluate the *in vitro* bioaccessibility and bioavailability of Arsenic (As), Cadmium (Cd) and Lead (Pb) in mineral clay samples collected from the Sierra Mountains (USA) using the Unified Bioaccessibility Research Group of Europe (UBM) method and the Caco-2 permeability assay, respectively. After UMB-gastric (UBM-G) digestion, As and Pb bioaccessibility were lower compared to Cd and decreased further in the UMB-gastrointestinal (UBM-GI) assay. Bioavailability estimates using the Caco-2 cell showed very low to non-detectable permeability for all 3 heavy metals. Thus, while initial heavy metal ranged from 3.8–17 ppm, 0.024–0.061ppm, and 5.8–20 ppm for As, Cd, and Pb, respectively, the bioavailability for these metals was reduced to very low levels that followed: non-detectable values of As, <0.007ppm of Cd, and <0.1ppm of Pb. Using UBM-digestion to mimic bioaccessibility, followed by Caco-2 cell bioavailability enabled us to conclude that *in vitro* assessment of heavy metal exposure associated with mineral clay-based natural health products does not pose a potential hazard to consumers.

## Introduction

Commercially available mineral clay products are widely used by consumers to relieve joint pain and muscle soreness, as well as a treatment for other chronic disease^1,2^. Mineral clay acidic extracts, in particular, are known to suppress the production of nitric oxide which is an important mediator for inflammatory reactions that cause the thinning of cartilage tissue^3^. Several clinical studies also report that naturally found mineral clays have the potential to improve overall joint health and function well in mild to moderate cases of osteoarthritis (OA) in patients^3–6^. It is to be noted that, OA is a major cause of disability in elderly populations around the globe^7^. In Canada, in particular; approximately 0.9% of Canadian adults (e.g. more than 272,000 people) suffer from OA, which translates to a substantial cumulative economic burden that has an estimated cost of $195.2 billion^8^. The hydrothermal mineral complex obtained from the Sierra Mountains in the United States is one such source of therapeutic clay. These clay products are rich in essential minerals, but also include potentially harmful heavy metals such as Arsenic (As), Cadmium (Cd) and Lead (Pb) at very low concentrations. Data from initial trials have also shown that depending on the ingestion dose, the levels of As and Pb present in these mineral clays could exceed current Health Canada’s tolerance limits, established for heavy metals present in natural health products^9^. Hence, despite having an important health benefit for OA patients, the total toxic heavy metal content in such mineral clay products that is bioaccessible and bioavailable needs to be evaluated to ensure the safety of these products.

In this study, we use the term heavy metal “bioaccessibility” to describe the fraction of heavy metals that is released from a mineral matrix into the gastrointestinal tract upon digestion; while the term “bioavailability” refers to the proportion of heavy metals that are absorbed from the intestine to enter the systemic circulation and thus available to induce a potentially toxic effect^10,11^. For our purposes, bioaccessibility was determined by the maximum soluble concentration of metals that were released from the sample clay mineral matrix using a synthetic oral, gastric and intestinal medium, equipped with enzymes to simulate gastrointestinal digestion. Factors such as metal species and speciation, as well as organic or inorganic components that co-exist in the matrix and which may act to sequester metals are known factors that will influence the extent of metal bioaccessibility^12,13^. Further risk assessment can be made by conducting *in vitro* bioavailability of the heavy metals following digestion using cultured Caco-2 cells, which assesses transfer behaviour and uptake of heavy metals^14,15^. Bioaccessibility and bioavailability assessment of heavy metals have formally been conducted to assess exposure from the soil, and water samples; agricultural crops and commodities such as, vegetables, seafood and natural health products^14–19^. The linear correlation reported between *in vitro* bioaccessibility assay for heavy metals and *in vivo* bioavailability data, reinforces its practical use to predict the exposure of heavy metals to humans^16,20–22^. The procedure also enables an alternative to animal testing methods that require protocals approved by animal ethics, and a need for rodent animal infrastructural facilities, whereas, in comparison, *in vitro* methods are less expensive, and rapid to complete.

Several standard methods are available to measure heavy metal bioaccessibility in mineral clay samples. Most often employed methods include the one-step USEPA method 1340; two-step methods that include the physiologically based extraction test (PBET)^23^ and the *in vitro* gastrointestinal method (IVG)^22^; and a three-step, Unified Bioaccessibility Research Group of Europe Method (UBM)^18,19^. The Bioaccessibility Research Group of Europe (BARGE) has developed UBM with the aim of producing a validated and standardized procedure to test heavy metal toxicity in the soil matrix^24^. The BARGE method has also been used to determine metals availability in fish and crab food sources^25^. The simulation consists of two phases; the gastric and gastrointestinal phase, where the gastric phase has samples treated with fluids that mimic the stomach fluid and the gastrointestinal phase where samples are digested with enzymes that simulate gastric and small intestine combined^19,24^. The USEPA method was initially established to measure Pb in soil samples and the same has never been tested on other matrices, including water, food and natural health products. Nevertheless, it should be noted that using different test methods will likely result in different estimates of bioaccessibility, due to a variety of variables that include medium pH, the composition of digestion solution, the sample to solution ratio, and digestion time^16,17^.

After being released from the mineral matrix and becoming bioaccessible, there are numerous factors that can subsequently influence the bioavailability of mineral components. For example, factors such as solubility, interactions with other dietary ingredients, molecular transformations, presence or absence of cellular transporters, metabolism and the interaction with the gut microbiota, all can potentially influence the level of bioavailability^26^. Recently, cell culture has been used extensively as an *in vitro* method to assess the bioavailability of minerals present in natural health products. The Caco-2 cell, a human colon adenocarcinoma cell line, has numerous morphological and biochemical characteristics that produce functional similarities to the small intestine when fully differentiated^27–33^. For these reasons, the Caco-2 cell has been shown to have usefulness to assess the *in vitro* bioavailability of heavy metal components in food products following gastric or gastrointestinal digestion^15^.

The current work seeks to evaluate the relative bioaccessibility of As, Cd and Pb in mineral clay products using UBM and USEPA methods and to further estimate the *in vitro* bioavailability of these heavy metals using a Caco-2 monolayer permeability assay. This combination of bioaccessibility followed by bioavailability assessment will facilitate a greater understanding of factors that will impact on risk assessment potential of natural health products which contain therapeutic clay.

## Results and Discussion

### Total As, Cd, and Pb in clay samples

The total As, Cd, and Pb concentrations in 10 batches of mineral clay samples are presented in Table 1. A wide range of heavy metal concentrations that included As (4–17 ppm); Cd (24–61 ppb) and Pb (6–20 ppm) were found. Results showed that As and Pb contents in clays collected from different sites (Sample 1–5 *versus* Sample 6–10) were quite variable. Indeed, factors specific to the sampling sites, such as geographical or environmental conditions, are often a major reason for high variability in heavy metal contents in soil and clay samples^34,35^. Moreover, variation in total heavy metal content, specifically for Pb, was also observed from different collecting times made at the same site (Sample 1–2 and Sample 3–5); an observation that was not made for samples that contained As and Cd, respectively, collected from the same site, but at different times. We have no actual explanation for this difference at this time, except to speculate that relative differences in pH of individual clay aggregates sampled herein could have been a factor since low pH (e.g. pH < 5) characteristics are known to correspond to greater solubility of lead salts^12^.

**Table 1:** Total As, Cd, and Pb content in mineral clay (ppm; mean ± SD).

Sample	As	Cd	Pb
1	15 ± 3.0	0.038 ± 0.013	20 ± 2.00
2	15 ± 1.0	0.040 ± 0.011	18 ± 1.00
3	17 ± 2.0	0.024 ± 0.005	6.1 ± 0.40
4	16 ± 3.0	0.034 ± 0.003	5.8 ± 0.90
5	16 ± 2.0	0.037 ± 0.020	6.4 ± 1.00
6	4.1 ± 1.0	0.060 ± 0.010	8.48 ± 1.09
7	3.8 ± 0.2	0.053 ± 0.012	8.52 ± 1.21
8	3.8 ± 0.3	0.052 ± 0.006	8.05 ± 0.19
9	3.8 ± 0.2	0.061 ± 0.002	8.73 ± 0.56
10	4.1 ± 0.4	0.053 ± 0.021	8.45 ± 0.28

From the results presented in Table 1 and the recommended dosage of most commercially available mineral clay products (11–15 mg per lb body weight per day), we estimated that our results translate to ingesting a maximum of 45 μg/day of As, 0.16 μg/day of Cd and 52 μg/day of Pb for a 175-lb person if 100% is bioaccessible and bioavailable (Table 2). At these levels, As and Pb, respectively, in mineral clay samples, are above the acceptable limit established by Health Canada, which prescribes a limit of 10 μg/day of As; 6 μg/day of Cd and 10 μg/day of Pb, respectively, as acceptable limits for these metals in natural health products^9^. It is of interest, however, that the intakes mentioned above are lower than those reported in FDA regulations for heavy metals in seafood^36^, wherein the agency identified a “tolerable daily intake” for organic As to be 130 μg per day and for Cd of 55 μg per day, and a “provisional tolerable total intake level” for Pb to be 75 μg/day.

**Table 2:** Bioaccessibility (%) of As, Cd, and Pb measured by UBM and USEPA methods.

Samples	USEPA			UBM Method					
				UBM-G			UBM-GI		
	As	Cd	Pb^d^	As^a^	Cd	Pb^e^	As^b^	Cd^c,g^	Pb^f^
1	6.62 ± 1.20	61.53 ± 09.54	15.24 ± 01.39	9.2 ± 1.2	63.1 ± 8.1	9.1 ± 0.3	9.1 ± 1.4	1.5 ± 0.1	1.4 ± 0.5
2	6.96 ± 0.27	80.01 ± 12.98	18.51 ± 01.20	9.8 ± 1.4	67.2 ± 13.1	13.3 ± 1.2	9.1 ± 1.3	1.9 ± 0.4	1.1 ± 0.3
3	7.89 ± 0.23	62.91 ± 10.89	1.43 ± 0.21	9.0 ± 1.2	47.1 ± 13.9	0.2 ± 0.2	8.8 ± 1.2	n.a.	n.a.
4	7.08 ± 0.39	52.92 ± 10.95	1.99 ± 0.45	7.9 ± 1.2	39.3 ± 4.7	n.a.	7.9 ± 1.6	n.a.	n.a.
5	5.65 ± 0.05	52.63 ± 07.93	0.88 ± 0.23	9.1 ± 1.5	45.3 ± 7.7	n.a.	8.5 ± 0.9	n.a.	n.a.
6	8.03 ± 0.17	42.52 ± 05.84	1.73 ± 0.04	11.8 ± 2.4	44.2 ± 15.0	6.7 ± 1.0	9.4 ± 0.5	9.0 ± 1.2	n.a.
7	8.33 ± 1.02	54.40 ± 11.45	2.37 ± 0.08	12.2 ± 2.0	56.7 ± 11.7	3.6 ± 1.1	10.2 ± 1.3	16.8 ± 1.7	n.a.
8	8.04 ± 0.43	53.76 ± 10.12	2.23 ± 0.03	12.2 ± 2.2	55.8 ± 5.5	2.4 ± 1.5	10.3 ± 1.4	6.3 ± 0.2	n.a.
9	7.09 ± 0.58	52.65 ± 11.24	2.20 ± 0.56	11.7 ± 1.7	50.9 ± 4.0	2.0 ± 0.2	9.0 ± 1.2	6.2 ± 0.6	n.a.
10	7.25 ± 0.92	69.12 ± 05.21	2.14 ± 0.08	10.9 ± 3.1	51.5 ± 13.3	2.1 ± 0.3	9.4 ± 1.1	5.7 ± 1.2	n.a.

Data are expressed as mean ± standard deviation.

^*^Bioaccessibilities of As, Cd, and Pb extracted with UBM-G method are significantly (P < 0.05) greater than that of UBM-GI methods.

^a,b^Bioaccessibility of As extracted with UBM-G and UBM-GI methods is significantly different (P < 0.05) between samples obtained from site 1 and site 2.

^c^Bioaccessibility of Cd extracted with UBM-GI methods is significantly different between (P < 0.05) samples obtained from site 1 and site 2.

^d–f^Bioaccessibility of Pb extracted with different methods is significantly different (P < 0.05) between site 1 samples obtained from different time periods.

^g^Bioaccessibility of Cd extracted with UBM-GI methods is significantly different (P < 0.05) between site 1 samples obtained from different time periods.

^n.a^minerals are lower than detection limit, therefore not able to be detected.

### Bioaccessibility of As, Cd, and Pb using USEPA method

In addition to determining the total heavy metal content in a formulated natural health product, obtaining information on the actual fraction of the heavy metals that are bioaccessible is required for risk assessment. This step involves elucidating the extent of release of heavy metals from clay samples after digestion and an accurate measurement of the metal in the digested fraction that is available to be absorbed.

The percent bioaccessibility of As, Cd, and Pb in mineral clay samples using the USEPA method is presented in Table 2. The results show some similarity to bioaccessibility data generated using the UBM methods, where Cd had relatively higher bioaccessibility compared to both As and Pb, respectively. It is of interest that we found that the bioaccessibility of Pb collected on from Site 1 in August 2011 was greater (P < 0.05) than those collected in February 2013. One explanation for this observation could be the difference in total metal content obtained from these particular samples. Looking closely at the As results, we note that As content was considerably higher (P < 0.05) in Site 1 samples, compared with Site 2 samples, but there was no significant difference between the bioaccessibility of As from both sites. A different observation was obtained for the concentration of Pb at both sites which also produced significantly different Pb bioaccessibility results (P < 0.05). Hence, although the bioaccessibility of As was not affected by the original concentration present, the same observation was not found with the Pb results. We suggest that factors that influence the bioaccessibility of minerals could be specific to the heavy metal of interest. Arsenic and bioaccessibility after USEPA digestion was <9% and <16%, respectively, while the bioaccessibility for Cd was much higher, ranging from 42 to 80%. This translates to the 175-lb person, mentioned in the example above, having a maximum of 3.6 μg/day of As, 0.10 μg/day of Cd and 8.75 μg/day of Pb available for absorption (Table 3). This result is within the maximum tolerable bioaccessible limit set by Health Canada^9^ for these particular heavy metals.

**Table 3:** Maximum concentration of available heavy metal allowed for absorption as based on the recommended daily dose of mineral clay samples^9^.

Samples	Initial total heavy metal content^a^ (μg/day)			USEPA^a^(μg/day)			UBM^a^(μg/day)					
	As	Cd	Pb	As	Cd	Pb	UBM-G			UBM-GI		
							As	Cd	Pb	As	Cd	Pb
1	40	0.099	52	2.6	0.061	8.00	3.68	0.062	4.70	3.6	0.001	0.7
2	40	0.105	48	2.7	0.084	8.75	3.92	0.070	6.40	3.6	0.002	0.5
3	45	0.063	16	3.6	0.040	0.23	4.05	0.030	0.03	4.0	n.a.	n.a.
4	42	0.090	15	3.0	0.047	0.30	3.32	0.035	n.a.	3.3	n.a.	n.a.
5	42	0.097	17	2.3	0.051	0.14	3.82	0.044	n.a.	3.5	n.a.	n.a.
6	11	0.157	22	0.8	0.067	0.39	1.30	0.067	1.48	1.0	0.014	n.a.
7	10	0.139	22	0.8	0.076	0.53	1.22	0.079	0.80	1.0	0.023	n.a.
8	10	0.136	21	0.8	0.073	0.47	1.22	0.076	0.50	1.0	0.009	n.a.
9	10	0.160	23	0.7	0.084	0.50	1.17	0.081	0.46	0.9	0.010	n.a.
10	11	0.139	22	0.8	0.100	0.48	1.20	0.071	0.47	1.0	0.008	n.a.

^a^Heavy metal available (μg/day) to a to 175 lb person before and after digestion (based on the recommended dose of 11–15 mg mineral clay/ lb body/day).

### Bioaccessibility of As, Cd, and Pb using UBM method

The total As, Cd and Pb concentration in extraction fluids were measured, and contents in the preparation blanks were calculated based on the concentration in the extraction fluids. Saliva, gastric, bile and duodenal fluids contained 0.078, 0.917, 0.654, 1.272 ng/mL As, respectively. Similarly, Pb content in the same digestion fluids were 0.347, 0.575, 0.446 and 0.236 ng/mL, respectively. These results corresponded to 0.017 and 0.066 μg/g As, and 0.036 and 0.056 μg/g Pb in the gastric and gastrointestinal digestion fluids, respectively. Cadmium was not detectable in the extraction fluids. Unlike the results obtained from the USEPA method, total As content was higher in Site 2 compared to Site 1 using both UBM digestion methods (Table 2). The underlying reasons for this observation is likely attributed to the differences in geographic conditions, which can impact the extraction efficiencies for different heavy metals. However, we did determine that in general, the bioaccessibility of As, Cd, and Pb after gastric digestion was higher than that obtained with gastrointestinal digestion; Cd and Pb showing a much greater difference compared with As. This difference can be attributed to the acidic conditions used for gastric digestion, which not only enabled the release of the minerals from the clay matrix but also provided the low pH required for optimal solubility of metal ions. Our result indicates that bioaccessibility of As reached around 10% with the gastric digestion, while the bioaccessibility for Cd was much higher, ranging from 40 to 70%. Hence, using the UBM-G method, the same 175-lb person model would require a maximum intake of 4, 0.08, and 6.4 μg/day, respectively (Table 3) for As, Cd and Pb in order to be bioavailable. These estimates meet the maximum tolerable limit for these heavy metals set by Health Canada^9^.

A different result was obtained using the UBM-GI assay, where the bioaccessibility of Cd and Pb decreased dramatically in the gastrointestinal digestion phase (<17% of Cd, <1.5% of Pb), compared to that observed using only gastric digestion. Lower bioaccessibility of these Cd and Pb metals corresponded to the increased pH conditions (pH 8.0), which were used to mimic the gastrointestinal digestion, compared to the low pH gastric digestion. The lower apparent bioaccessibility of these metals from the clay matrix is attributed to the poor solubility at neutral to alkaline conditions. This was not observed for As using the UBM-GI bioaccessibility digestion, where little change occurred and recovery remained at approximately 10%.

Hence, the maximum bioaccessibility of 4 μg/day for As, 0.02 μg/day for Cd, and 0.7 μg/day for Pb (Table 3) when translated to our 175-lb subject is within the tolerable limits, according to Health Canada regulations^9^. Former studies conducted by other researchers using medicinal clay samples (Natural Health Product)^37,38^ reported similar results. It should also be noted, that the total heavy metal concentrations in many of these former studies were also reduced by ~99% after gastric/gastrointestinal digestion. Koch *et al*.^37^ demonstrated that only 4% of the total As content in “Niu Huang Jie Du Pian pills (Natural Health Supplement)” was bioaccessible after UMB-GI digestion. They also validated their findings by measuring heavy metal excretion in urine. More recently, bioaccessibility measure of As content in 42 medicines, reported only 12 preparations were bioaccessible, and only one had a bioaccessible total inorganic As concentration that exceeded the safe limit (USEPA Oral RfD)^38^.

### Comparison of bioaccessibility of As, Cd, and Pb using UBM and USEPA method

The UBM method is an internationally recognized method for testing heavy metal bioaccessibility in soil matrices, but it has not yet received acceptance for similar use by Health Canada’s Natural and Non-prescription Health Products Directorate (NNHPD) for assessing heavy metal bioaccessibility in natural health and food products^9^. The NNHPD recommends either the use of acid hydrolysis (with a strong acid such as HNO_3_) followed by measurements on ICP-MS or ICP-AES, or a colorimetric method (e.g. FCC) for detecting heavy metals in finished natural health products. Similar approaches are approved by other regulatory agencies for heavy metal testing in natural health products^23,39^. The UBM method is based on the concept that metals bound in different soil types that have a mineral matrix will solubilize differently in the GI tract, thus influencing bioavailability. Only the soluble or the bioaccessible fraction of the metal in the GI tract is made available for absorption before ultimately reaching the systemic circulation^19^. The non-soluble fraction of the metal is expected to be excreted in its bound form in the feces. Although researchers have indicated that this method could be used for an exposure assessment in food products such as fruits, vegetables and natural health products^25,40^.

Our results indicate that Cd bioaccessibility using the USEPA method agreed with the UBM-G analysis which reflects only the gastric digestion phase. The limitation of using the USEPA method to predict heavy metal bioaccessibility from natural health products taken orally is that it is a generic single-step extraction procedure, while, UBM-GI simulates digestion from gastric and gastrointestinal phases. Based on these observations, we conclude that the UBM-GI method is a better procedure to estimate bioaccessibility since it more closely mimics the conditions of the human GI tract. The lower solubility of Cd and Pb due to the alkaline conditions present with the total gastrointestinal digestion method apparently also lead to lower absolute values for bioaccessibility. When extrapolating this observation to an *in vivo* situation, the insoluble heavy metals, such as Cd and Pb, would not be expected to be free for absorption and thus excreted in feces. Other factors to consider affecting bioaccessibility of Cd and Pb from clay samples could be such factors as the presence of phytochelatins, metal species and its speciation^13,15^.

### Bioavailability of As, Cd, and Pb

Prior to conducting the Caco-2 permeability assay, we determined the potential toxicities of the heavy metal preparations using MTT-redox assay for cell viability and relative epithelial resistance (%, RES) to evaluate the effect of mineral clay digest on the integrity of cell monolayers. MTT results were greater than 90% control indicating that no toxicity existed at the concentrations used in this experiment. Similarly, the TEER value for all samples tested in this study was greater than 90%, thus indicating that the mineral clay digest did not affect the integrity of Caco-2 monolayer.

**Figure 1:**
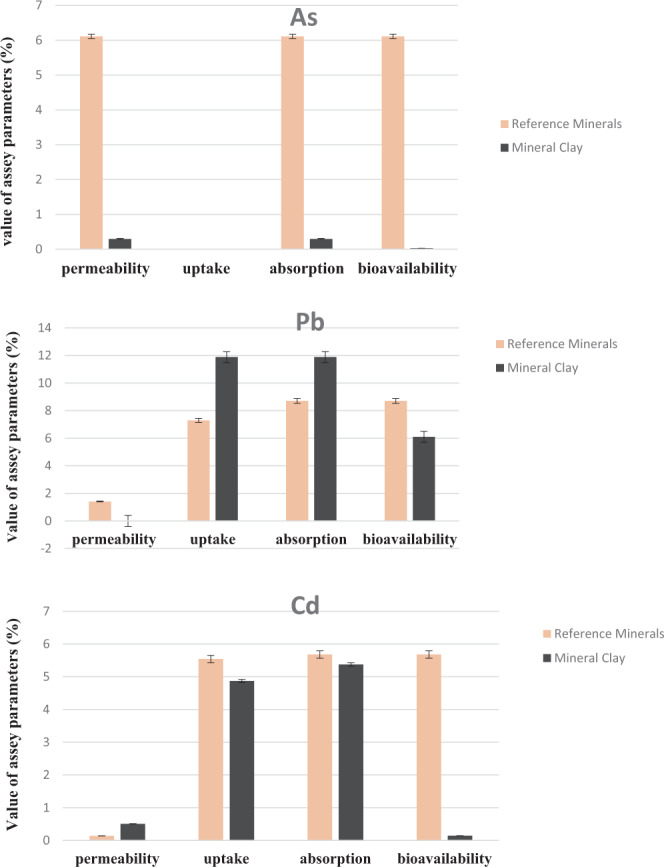
Representation of bioavailability (%) of As, Pb and Cd in reference mineral and mineral clay. Measures of bioavailability are mean values derived in triplicate from a single Caco-2 cell culture. A total of 3 experiments were performed all showing similar trends in terms of individual metal transport characteristics.

Figure 1 shows the bioavailability of As, Cd, and Pb in both reference form as well as from mineral clay digest samples, respectively. Very low permeability values obtained for As, Pb and Cd were equivalent to 0.295%, 0.078%, and 0.506%, respectively. The uptake of As was not detectable in all samples whereas, for Pb and Cd the values were <12% for all samples. Overall, the bioavailability of Cd was 6.1%, while for Pb it was 0.144%. Previous studies have shown that bioavailability of Pb in the mineral matrix is dependent on its association with carbonate complexes which enhances bioaccessibility^17^. Mineral analysis of clay samples has listed the presence of many divalent ions, such as calcium, magnesium, and iron, which are known to immobilize arsenate and lower its solubility^13^. Another parameter to consider for bioaccessibility is the residence time of the matrix in the gastric compartment. The greater the residence time of the matrix in the gastric compartment, the more efficient the extraction. Overall, our results indicated that the bioavailability of As, Cd, and Pb in mineral clay samples ranged from very low to non-detectable levels, which suggests a low risk to human health.

### Correlation between bioaccessibility and bioavailability

In order to use *in vitro* bioaccessibility assays (such as UBM and USEPA) as a surrogate measurement of heavy metal dietary exposure, other works have used correlations to show the extent of agreement between ‘*in vitro* bioaccessibility *and in vitro* bioavailability assays^20,41,42^. This approach has been used by researchers interested in various oral preparations that include heavy metals in mineral clay complexes and nutrients in food or natural health products^22,43,44^. In the present study, we present correlations between different bioaccessibility essays (UBM-G *vs* UBM-GI and UBM *vs* USEPA) to interpret bioaccessibility results derived from different assay methodology. The bioaccessibility of As using UBM-GI was significantly correlated with UBM-G (r = 0.865, P < 0.001). However, no correlation was established for Cd and Pb using the UBM gastric and gastrointestinal digestion methods. This was attributed to the very low to non- detectable bioaccessibility values obtained for Cd & Pb concentrations using the UBM-GI assay.

On the other hand, As bioaccessibility measured by the USEPA method was correlated with the UBM-G (r = 0.601, P < 0.01) and UBM GI (r = 0.643, P < 0.01) assays. We conclude therefore that there is good potential for using the BARGE method for estimating heavy metal detection of mineral clay products when prepared for gastrointestinal digestion. We also examined if correlations existed between bioaccessibility (UBM-G) and bioavailability, using Caco-2 cell methodology. Cadmium bioaccessibility was significantly (r = 0.798, P < 0.01) correlated with *in vitro* bioavailability, measured using the Caco-2 monolayer assay. This was not the case for Pb, which we attribute to the fact the bioavailability values of Pb were non-detectable due to poor solubility.

## Conclusion

The results of our comparison of using three different bioaccessibility assays, showed that only 10–16% of the original As and Pb content present in mineral clay complexes was free for absorption. On the other hand, the bioaccessibility values for Cd were relatively high; however, it should be noted that the initial Cd content in the clay samples was comparatively low (24–61ppb). This therefore equates to 0.16 μg/day of Cd consumption by a 175-lb adult consuming maximum prescribed doses of mineral clay product. This estimate shows that the initial Cd content in mineral clay is below the total minimum Cd limit established by Health Canada (<6.0 µg/day). The UBM and USEPA bioaccessibility methods were compared, and the UBM method was found to relatively more effective in mimicking the human stomach digestion process on this product matrix. Based on these observations, we support using the UBM method for heavy metal bioaccessibility in natural health products.

Extending this study to include the bioavailability of these heavy metals using the Caco-2 monolayer cell culture assay revealed that all metals, after correction for bioaccessibility, produced very low to non-detectable permeability estimates. It can be concluded from these combined in- vitro bioaccessibility and bioavailability results, that a very low probability of risk associated with consumption of these mineral clays exists if the recommended usage specifications (11–15 mg per lb body weight) is followed.

## Methods

### Materials

All reagents used were of analytical grade. All solutions were prepared using ultra-high pure (UHP) 18 Ω water. All laboratory ware was soaked for 24 h in an acid bath containing 10% (v/v) nitric acid and then rinsed with UHP water. Concentrated hydrochloric acid (12 mol L^−1^), HNO_3_ 69%, NaCl, NH_4_Cl, anhydrous Na_2_SO_4_, CaCl_2_.2H_2_O, NaHCO_3_, anhydrous D ( + )-glucose, sodium arsenate dibasic heptahydrate, cadmium acetate and lead acetate trihydrate were obtained from Fisher Scientific. d-glucuronic acid, Pancreatin (pig), pepsin (pig), Bovine serum albumin (BSA), KSCN, NaH_2_PO_4_, mucin (pig), D-glucosamine hydrochloride, lipase (pig), α-amylase (*Bacillus species*), urea and bile salts (bovine), modified eagle medium (DMEM) containing 4.5 g/L glucose, penicillin-streptomycin solution (10, 000 units penicillin and 10 mg streptomycin per mL), and Hank’s Balanced Salt Solution (HBSS) and glycine were obtained from Sigma–Aldrich (St. Louis, MO, USA). KH_2_PO_4_, MgCl_2_·6H_2_O, KCl, and uric acid were obtained from VWR. Caco-2 cells (HTB-37™) were purchased at passage 18 from the American Type Culture Collection (ATCC, Manassas, VA, USA). Fetal bovine serum and trypsin-EDTA were purchased from Invitrogen (Burlington, ON, Canada).

### Collection of mineral clay samples

The clay samples were collected at different times from 2 locations at Sierra Mountains (USA). The specific sample details (year, month, location) are shown in Table 4. The clay samples were air-dried, disaggregated sieved (2 mm) to remove large pieces, and then screened to exclude particles greater than 250 μm. Samples were kept at room temperature in a sealed container until three-phase (saliva/gastric/gastrointestinal) *in vitro* digestion.

**Table 4:** Sample information of mineral clay.

Sample No.	Sample collection Site	Sample Code	Month	Year
1	Site -1		August	2011
2			August	2011
3			February	2013
4			February	2013
5			February	2013
6	Site -2		October	2014
7			October	2014
8			October	2014
9			October	2014
10			October	2014

### Instrumentation

Elemental analysis was carried out using an inductively coupled plasma mass spectrometer (ICP-MS, Agilent 8900 Triple Quadrupole (QQQ) ICP-MS) coupled to a cross-flow nebulizer. The operating parameters of the ICP-MS instrument were as follows: Sampling/skimmer cones = Pt; RF power =1100 W; Signal measurement = Peak Hopping; Detector voltage = Pulse 1250 V; Gas flow rate; Main 15.0 - L/min; Auxiliary - 1.2 L/min; Nebulizes - 1.0 L/min. These measurements were converted to μg/L by referencing a calibration curve.

### Total heavy metal content of original clay sample

Total As, Cd and Pb concentrations in clay samples were analyzed using ICP-MS after digestion with HNO_3_^45^. Approximately 0.2 g of mineral clay samples were mixed with 6 ml of HNO_3_, 2 ml of HCL and 2 ml of hydrofluoric for the simultaneous extraction of metals. The solution was digested by microwave using the following procedure: heated to 120 °C in 8 minutes and holding 3 min; raising the temperature to 150 °C maintaining 5 min; increase the temperature to 190 °C keeping 35 min. After cooling, 2 ml of H_2_O_2_ was added to the digested mixture then heated at 140 °C in a heating block until the residue solution left about 1 mL. Finally, the solution was transferred into 50 volumetric flasks, brought to volume with water and mixed fully. The determination of metals was performed by ICP-MS with internal standard method and standard addition method. Multielement Calibration Standard 3 diluted to 10 mg/L (in 5% HNO3) was used for instrument calibration. Minimal detection limits for As, Cd and Pb were, 0.1 μg/L; 0.01 μg/L; 0.02 μg/L, respectively.

### *In vitro* bioaccessibility using USEPA method

Bioaccessibility measurements of heavy metals using the USEPA method were performed according to details outlined in the USEPA method 1340^46^. Mineral clay samples (10 batches of samples, duplicate, 1.0 g each) were weighed into high-density polyethylene (HDPE) bottles containing 100 mL extraction fluid; consisting of 0.4 mol/L glycine (free base, reagent-grade glycine in deionized water), adjusted to a pH of 1.50 ± 0.05 using 0.5% HCl. Samples were pre-heated to 37 ± 2 °C followed by extraction, which involved rotating the samples at 30 ± 2 rpm for one hour. An aliquot (40 mL) of the supernatant was filtered through a 0.45μm cellulose filter followed by heavy metal analysis using ICP-MS as described above.

### *In vitro* bioaccessibility measurement using UBM method

#### Preparation of gastric and intestinal solutions

Gastric and gastrointestinal fluids were prepared according to the procedure described by Wragg *et al*.^19^ To prepare 1000 ml of simulated saliva fluid, amylase (145 mg), mucin (50 mg), and Uric Acid (15 mg) were added in a 2 L HDPE screw top bottle. Simultaneously the inorganic (500 ml) and organic saliva phase (500 ml) reagents were added together and mixed thoroughly^19^. The pH of the simulated saliva fluid was adjusted to 6.5 ± 0.5. To prepare the simulated gastric fluid (1 L, Bovine Serum Albumin (1000 mg), mucin (3000 mg) and pepsin (1000 mg) were added together into a 2 L HDPE screw-top bottle, and mixed thoroughly. The pH of the simulated saliva fluid was adjusted to 1.0, using HCL. To prepare 1 L simulated duodenal fluid, CaCl_2_ (200 mg), bovine serum albumin (1000 mg), pancreatin (3000 mg) and lipase (500 mg) were added in a 2 L HDPE screw-top bottle and mixed thoroughly. The pH of the simulated duodenal fluid was adjusted to 7.4 ± 0.2 using 0.5 M NaOH. The bile fluid (1 L) was prepared by adding CaCl_2_ (222 mg), bovine serum albumin (1800mg) and bile (6000 mg) to a 2 L HDPE screw-top bottle and mixed thoroughly^19^. The pH of the simulated duodenal fluid was pH 8.0 ± 0.2 and the final pH of gastric and gastrointestinal phases were 1.2–1.4 and 6.3 ± 0.5, respectively.

#### Determination of heavy metal bioaccessibility using UBM digestion method

The digestion procedures were performed based on the method described by Wragg *et al*.^18^ Briefly, for gastric phase digestion, samples of mineral clay (10 batches of 0.6 g samples in duplicate) were mixed with 9.0 mL of saliva solution (pH 6.5 ± 0.5) in 50 mL falcon tubes and shaken manually for 30 s; followed by mixing with 13.5 mL of gastric solution (pH 0.9–1.0) using a rotator set at 30 ± 2 rpm for 1 h in a 37 °C incubator. This represented the gastric phase fluid, and heavy metals were analyzed from this fluid after centrifugation at 4500 × g for 15 minutes and a 10 times dilution with 1% HNO_3_ (v/v) using ICP-MS. For the gastrointestinal phase, an additional 9 mL of simulated bile fluid (pH 8.0 ± 0.2) and 27 mL of simulated duodenal fluid (pH 7.4 ± 0.2) were added to the gastric phase fluid. The mixture was shaken at 30 ± 2 rpm for another 4 h in a 37 °C incubator and then centrifuged at 4500 × g for 15 minutes, followed by 10 times dilution with 0.5% HCl (v/v). Final samples were then analyzed for heavy metal bioaccessibility using ICP-MS.

Ten clay samples were digested and analyzed using the method described above. The bioaccessibility of metals present in mineral clay samples are defined as:

$$Bioaccessibility( \% )=\frac{Metal\,content\,in\,aqueous\,phase}{Metal\,content\,in\,original\,sample\,}\times 100$$

$$Bioaccessible\,content\,(ppm)=Initial\,heavy\,metal\,in\,clay\,(ppm)\times \frac{Bioaccessibility\,( \% )}{100\,}$$

### *In vitro* bioavailability measurement using Caco-2 cells

#### Caco-2 cell culture

The *in vitro* bioavailability of heavy metals was analyzed using Caco-2 cell culture^33,47^. Briefly, Caco-2 cells (HTB-37, ATCC) were cultured in Dulbecco’s Modified Eagle Medium (DMEM) containing 4.5 g/L glucose (Sigma, St. Louis, MO, USA) supplemented with 10% fetal bovine serum (FBS) (Invitrogen, Canada), 100 µg/ml of penicillin and 100 µg/ml of streptomycin at 37 °C under a 5% CO_2_ atmosphere. The medium was changed every 2–3 days, and the cells were subcultured weekly by trypsin-EDTA treatment. Twenty-one day old, differentiated, Caco-2 cells were seeded onto 6-well translucent Transwell inserts (24 mm diameter, 0.4 µm pore size, high-density polyethylene terephthalate membrane, BD Biosciences, San Jose, CA, USA) at a density of 2.5 × 10^5^ cells/cm^2^ and allowed to grow for 3 weeks. All the cells used in this study were between 22 and 29 passages. The toxicity potential of heavy metal samples to Caco-2 cells was conducted using MTT redox assay^47^. Transepithelial electrical resistance (TEER) values were measured using a volt-ohmmeter (Millicell® ERS, Millipore, Bedford, MA, USA) to assess the integrity of the monolayers.

#### *In vitro* bioavailability measurement

Gastrointestinal digested solutions were boiled for 5 min to deactivate the enzymes followed by adjustment to pH 7.0 using 10 N NaOH. Sodium arsenate dibasic heptahydrate (As), cadmium acetate (Cd), lead acetate trihydrate (Pb) were used as references to test the bioavailability of free minerals. Samples were dissolved in 18 Ω H_2_O and diluted in HBSS to the concentration of 1, 0.1, and 1 μg/mL, respectively. The digested clay, and reference-heavy metal samples were sterilized through filtration followed by three times dilution in HBSS. For heavy metal permeability measurements, the culture medium in the insert or well was first removed and 1.5 ml of the diluted digested aqueous solution, or reference samples, were added to the apical side and 2.6 mL HBSS was added to the basolateral chamber. The cell cultures were incubated at 37 °C, 5% CO_2_ and 95% relative humidity for 2 h. An aliquot of the solution from the basolateral chamber was collected for heavy metal analysis. Caco-2 monolayers were then washed three times with phosphate-buffered saline (PBS) and incubated with 1 mL trypsin-EDTA to collect the cells. Cells were aspired and then centrifuged for 10 min at a speed of 1000 × g and digested with 1 mL concentrated HNO_3_ for 4 h at 110 °C and left overnight at room temperature. Then 2 mL of 18 Ω H_2_O was added into the digested cells. As, Cd and Pb present in Caco-2 cell digest and sampled from the bottom compartment of the insert, representing the basolateral solution were analyzed using ICP-MS according to the methods outlined above. Three experiments were conducted using Caco-2 cells prepared at the same stage of development and cell density and samples were tested in triplicate for each experiment. All three experiments showed similar trends for each measure of bioavailability. A representative result of the three experiments was presented.

Parameter measurements used to assess the bioavailability of heavy metals present in mineral clay samples are defined as:

$$Permeability\,( \% )=\frac{Heavy\,metal\,in\,basolateral\,chamber}{Heavy\,metal\,in\,aqueous\,phase\,added\,}\times 100$$

$$Uptake\,( \% )\,by\,cells=\frac{Heavy\,metal\,in\,cells}{Heavy\,metal\,in\,aqueous\,phase\,added\,}\times 100$$

$$Absorption\,( \% )=Permeability\,( \% )+uptake\,( \% )$$

$$Bioavailability\,( \% )=bioaccessibility\,( \% )\times absorption\,( \% )$$

$$Bioavailable\,content\,(ppm)=Initial\,heavy\,metal\,in\,clay\,(ppm)\times \frac{Bioavailability\,( \% )}{100\,}$$

Samples collected from bioaccessible fractions and monolayer Caco-2 cells were analyzed by ICP-MS.

### Statistical analysis

All assays were conducted at least in duplicate, and significant differences were established between means at P < 0.05. The different bioaccessibility (%) between sites were assessed using student’s t-test. Correlations were also determined using statistical tools (Minitab Statistical Software Version 16, Minitab Inc., State College, PA, USA).

## Data Availability

The datasets generated during and/or analyzed during the current study are available from the senior author on reasonable request.
